# Molecular epidemiology and drug sensitivity pattern of *Mycobacterium tuberculosis* strains isolated from pulmonary tuberculosis patients in and around Ambo Town, Central Ethiopia

**DOI:** 10.1371/journal.pone.0193083

**Published:** 2018-02-15

**Authors:** Melaku Tilahun, Gobena Ameni, Kassu Desta, Aboma Zewude, Lawrence Yamuah, Markos Abebe, Abraham Aseffa

**Affiliations:** 1 Armauer Hansen Research Institute (AHRI), Addis Ababa, Ethiopia; 2 Department of Medical Laboratory Science, Addis Ababa University, Addis Ababa, Ethiopia; 3 Aklilu Lemma Institute of Pathobiology, Addis Ababa University, Addis Ababa, Ethiopia; Indian Institute of Science, INDIA

## Abstract

**Introduction:**

Tuberculosis (TB) is caused by *M*. *tuberculosis* complex and remains a major global public health problem. The epidemic remains a threat to sub-Saharan Africa, including Ethiopia, with further emergence of drug resistant TB. We investigated the drug sensitivity pattern and molecular epidemiology of mycobacterial strains isolated from pulmonary TB patients in and around Ambo town in Oromia Region, Central Ethiopia.

**Methods:**

A cross-sectional study was conducted involving 105 consecutive new smear positive pulmonary TB patients diagnosed at Ambo Hospital and surrounding Health Centers between May 2014 and March 2015 upon informed consent. Sputum samples were cultured on Löwenstein-Jensen (LJ) media using standard techniques to isolate mycobacteria. Region of difference 9 (RD9)-based polymerase chain reaction (PCR) and spoligotyping was employed for the identification of the isolates at species and strain levels. The spoligotype patterns were entered into the SITVIT database to determine Octal and SIT (Spoligotyping International Typing) numbers for each strain. The sensitivity of the isolates to isoniazid (INH), rifampicin (RIF), ethambutol (ETB) and streptomycin (STM) was evaluated on LJ-medium with the indirect proportion method.

**Results:**

Cultures were positive in 86/105 (82%) of newly diagnosed smear positive pulmonary TB cases. All of the 86 isolates were confirmed as *M*. *tuberculosis*. The majority (76.7%) of them were clustered into seven groups while the rest (23.3%) appeared unique. The most predominant Spoligotypes were SIT53 and SIT149, consisting of 24.4% and 20.9% of the isolates, respectively. Assigning of the isolates to family using SPOTCLUST software revealed that 45.3% of the isolates belonged to T1, 23.3% to T3 and 13% to CAS family. The majority (76.7%) of the *M*. *tuberculosis* isolates were susceptible to all the four drugs. Any resistance to any one of the four drugs was detected in 23.3% of the isolates. The highest proportion of any resistance was observed against isoniazid (9.3%) and ethambutol (7%). There was only a single case (1.2%) of multidrug resistant/rifampicin resistant (MDR/RR) TB.

**Conclusion:**

The majority of the isolates were clustered suggesting on-going active transmission in the study area. Mono resistance is relatively prevalent while the magnitude of MDR/RR-TB was found to be lower than in previous studies.

## Introduction

TB, caused by *M*. *tuberculosis* complex, remains one of the world’s deadliest communicable diseases. In 2015, the World Health Organization (WHO, 2016) estimated that 10.4million people developed TB and 1.4million died from it. In addition to the TB epidemic, drug resistant TB has become a global challenge. In 2015, the prevalence of MDR/RR-TB was 3.9% among new cases worldwide [1]. African countries south of the Sahara, including Ethiopia, are heavily affected by TB. Along with 7 others from Africa, Ethiopia is one of the 30high burden countries for MDR-TB with an annual estimate of 3300 MDR/RR-TB among notified pulmonary cases in 2015 [1, 2].

At the molecular level, the global TB epidemic consists of multiple genotype-specific sub-epidemics resistant to the first line anti-TB drugs. Therefore, the knowledge of molecular epidemiology of MDR-TB is useful in supporting the TB surveillance and control program of countries. Early detection of drug resistant TB is critical to avoid further spread of the disease [3, 4]. Nevertheless, data on the molecular epidemiology of drug-resistant TB is still scanty in Ethiopia with only few studies performed in selected regions of the country such as by Tesema *et al*. who recently reported on the molecular epidemiology and transmission dynamics of *M*. *tuberculosis* in northwest Ethiopia, and described the gene mutations associated with isoniazid and rifampicin resistance among *M*. *tuberculosis* isolates [5, 6].

In recent years, drug resistance appears to be on the rise. There is a pressing need for extensive surveillance and establishment of advanced diagnostic facilities for early detection and expansion of second line treatment centers to prevent MDR-TB transmission [7]. According to the Federal Ministry of Health second national TB drug resistance survey report, the proportion of MDR-TB was2.3% of new cases; a significant increase from the first-round report of 1.6% [2]. In 2015, the estimate for MDR/RR-TB was 2.7% [1].

A pilot study had previously been conducted to generate preliminary information on the drug sensitivity pattern of a small number of *M*. *tuberculosis* isolates from Ambo area [8]. Because that preliminary study could not provide conclusive information, additional data were needed for use by the TB control program in the area. The present study was conducted to investigate into the epidemiology of TB in and around Ambo Town and to evaluate the drug sensitivity pattern of *M*. *tuberculosis* strains isolated from pulmonary TB cases in the study area.

## Methods

### Study settings

This cross-sectional study was conducted in and around Ambo Town, West Shoa Zone, central Ethiopia in Ambo Hospital, and in two nearby private health facilities (Agape Medium Clinic and Ambo Clinic). Ambo is located in the West Shoa Zone of the Oromia Region, west of Addis Ababa, Ambo Town has a latitude and longitude of 8°59′N 37°51′E and an elevation of 2101 meters. The study area includes the population in and around the major roadside urban center of Ambo town and the adjacent rural towns. Most of the diagnosis and all TB treatment in the area are provided through the DOTS TB clinics.

### Study population

Between May 2014 and March 2015, a total of 105 newly diagnosed smear positive adult pulmonary TB patients who gave written informed consent were enrolled in the study. Socio demographic data and clinical information were collected by trained health professionals using a pre-tested standard questionnaire. Data on HIV (Human Immunodeficiency virus) status was obtained from health facility records. The participants were requested to give three sputum samples (spot-morning-spot). Sputum smears were prepared and examined by an experienced facility laboratory technologist on site. The reading was systematic and standardized to scan at least 100 high power fields before producing a negative result. Sputum samples from acid-fast bacilli (AFB) smear positive patients were pooled individually into 50 ml sterile screw capped universal test tubes and stored at the diagnostic centers at -20°C for a maximum of five days until transported on a cold chain to the core laboratory at AHRI.

### Mycobacterial culture

Egg based Löwenstein-Jensen -pyruvate and Löwenstein-Jensen -glycerol media were prepared according to standard procedure [9]. All samples were processed and cultured using standard methods. Briefly, the sputum samples were decontaminated by equal volume of 4% sodium hydroxide solution for 15 minutes. The decontaminated samples were centrifuged at 3000 rpm for 15 min. The supernatant was discarded and a drop of phenol red solution was added into the supernatant as indicator and the sediment neutralized using 2N hydrochloric acid solution drop by drop. The sediment was inoculated into conventional Löwenstein-Jensen egg medium containing 0.6% sodium pyruvate and glycerol and incubated at 37°C for at least 8 weeks, with weekly observation for the presence of mycobacterial colonies. Microscopic examination of the colonies was performed using Ziehl-Neelsen staining method. Colonies from AFB positive isolates were collected into two cryo vial. One vial was used to prepare heat killed cells for molecular typing with bacteria treated at 80°C in a sonicator water bath for one hour. The other vial was stored at -80°C in freezing medium until sub-cultured for drug sensitivity test (DST).

### Region of difference 9 (RD9)-based polymerase chain reaction

Heat killed cells were used for polymerase chain reaction (PCR)-based deletion typing. The presence or absence of regions of difference-9 (RD9) was checked to identify *M*. *tuberculosis* from the other species of *M*. *tuberculosis* complex. The primers used for RD9 deletion typing were: RD9flankF, 5′-GTG TAG GTC AGC CCC ATC C-3′; RD9intR, 5′-CTG GAC CTC GAT GAC CAC TC-3′; and RD9flankR, 5′-GCC CAA CAG CTC GAC ATC-3′. PCR amplification of the mixtures was performed using a Thermal Cycler according to standard procedures [10]. Briefly, the reaction mixture was prepared and DNA amplified with the cycling condition of 10 min of enzyme activation at 95°C, 1 min of denaturation at 95°C, 0.5 min of annealing at 55°C,2 min of extension at 72°C for, involving a total of 35 cycles, and a final extension at 72°C for 10 min. The product was electrophoresed in 1.5% agarose gel in 1× triacetate- ethylene diamine tetra acetic acid (EDTA) running buffer. Ethidium bromide at a ratio of 1:10, 100 base pair (bp) DNA ladder, and orange 6× loading dye were used in gel electrophoresis and the gel was visualized using a Transilluminator (BIO RAD Laboratories Inc. www.bio-rad.com). Detection of a band size of 396 bp was considered as positive for *M*. *tuberculosis*, whereas detection of a band size of 575 bp was considered to be positive for the other members of *M*. *tuberculosis* complex species (*M*. *bovis* or *M*.*africanum*).

### Spoligotyping

Isolates confirmed to be *M*. *tuberculosis* using RD9 deletion typing were further identified by spoligotyping according to standard procedure [11]. The direct repeat (DR) region was amplified by a Thermal Cycler using oligonucleotides and primers derived from this region. The reaction mixture was amplified with PCR and the amplified product was hybridized to a set of 43 immobilized oligonucleotides, each corresponding to one of the unique spacer DNA sequences within the DR locus. After hybridization, the DNA was detected by the enhanced chemiluminescence and by exposure to X-ray film as specified by the manufacturer. The hybridization patterns were converted into binary and octal formats and compared with previously reported strains in the recent SITVIT database [12].

### Conventional drug susceptibility testing

The indirect proportion method was used with 7H10 medium on 24-well tissue culture plates (Becton Dickinson Company, USA). The DST protocol used in the study was the standard protocol of the TB laboratory of the Armauer Hansen Research Institute (AHRI) which itself was adopted from the guidelines of the World Health Organization [13]. The four anti-TB drugs (Isoniazid, Rifampicin, Ethambutol and Streptomycin) were mixed with the media at the recommended concentration and dispensed into 9 wells of the 24 well tissue culture plates and two wells were dispensed with drug free media. A standardized bacterial suspension was prepared and 10μl of the prepared bacterial suspension was dispensed into each of the drug containing and one drug free media. The remaining well with drug free medium was inoculated with 10μl of 1% (1:100) bacterial suspension. The plate was incubated in an inverted position at 35°C in the presence of sufficient humidity. Bacterial growth was checked on day 6, 12 and 19of culture. Resistance was expressed as the percentage of colonies that grew on critical concentrations of the substances, i.e. 0.2 μg/ml for isoniazid, 5μg/ml for ethambutol, 2μg/ml for streptomycin, and 1μg/ml for rifampicin. The interpretation was based on the standard criteria for resistance, i.e. 1% for all drugs [14]. For internal quality control, *M*. *tuberculosis* H37Rv strain sensitive to all anti-TB drugs was processed with the samples. Control strains resistant to the different anti-TB drugs were inoculated into the wells where the respective anti-TB drugs had been added.

### Data analysis

Socio-demographic and clinical data obtained through questionnaires and the results of laboratory tests were entered into SPSS version 20 data record files. Numerical variables such as age of patients and SIT numbers were entered as they were without being recoded. On the other hand, categorical variables like sex, marital status, history of hospital admission, HIV status, TB patient contact history, previous history of TB treatment, culture result and deletion typing and drug susceptibility pattern data were entered after being recoded. Statistical analysis was performed using SPSS software packages.

### Ethical issue

Ethical approval was obtained from Addis Ababa University School of Allied Health Sciences Department of Clinical Laboratory Science (DRERC) and AHRI/ALERT Ethics Review Committee. Support letters were obtained from the Oromia Regional Health Bureau and West Shoa Zonal Health Department. The purpose and benefit of the study was explained to each eligible study participant and questions answered. Those who were willing to participate in the study signed the informed consent form and were included in the study. All drug susceptibility test results were reported to the respective health facilities for further management of the patients. Furthermore, the confirmed MDR-TB case identified in this study was referred to the MDR-TB treatment center for further management.

## Results

### Socio demographic characteristics

The mean age of the study participants was 30.9 (+/- 11.4 SD) years and ranged from 18–67 years. The majority of the study participants (86%) were in the age group of 18 to 44 years. Half of the 86 culture positive study participants were male (male to female ratio of 1:1). Review of the marital status of the study participants showed that 59.3%, 38.3%, 1.2% and 1.2% were married, single, separated or widowed, respectively. Among the participants, 30.2% had history of contact with a TB patient in their household and 8.1% had a history of hospital admission. HIV status was known for only 58.1% of the participants. Among those tested, 16% (n = 50) were HIV positive (Table 1).

**Table 1:** Socio demographic characteristics and HIV status of study participants (n = 86) 2015.

Variables	Frequency in No (%)
**Age Group (years)**	
18–24	28(32.5)
25–34	30(34.9)
35–44	16(18.6)
45≥	12(14.0)
**Gender**	
Male	43(50.0)
Female	43(50.0)
**Marital Status**	
Single	33(38.3)
Married	51(59.3)
Separate	1(1.2)
Widowed	1(1.2)
**TB Patient Contact**	
Yes	26(30.2)
No	60(69.8)
**Hospital Admission**	
Yes	7(8.1)
No	79(91.9)
**HIV Status**	
Positive	8(9.3)
Negative	42(48.8)
Unknown	36(41.9)

### Genetic diversity and family assignment of bacterial isolates

**Fig 1:**
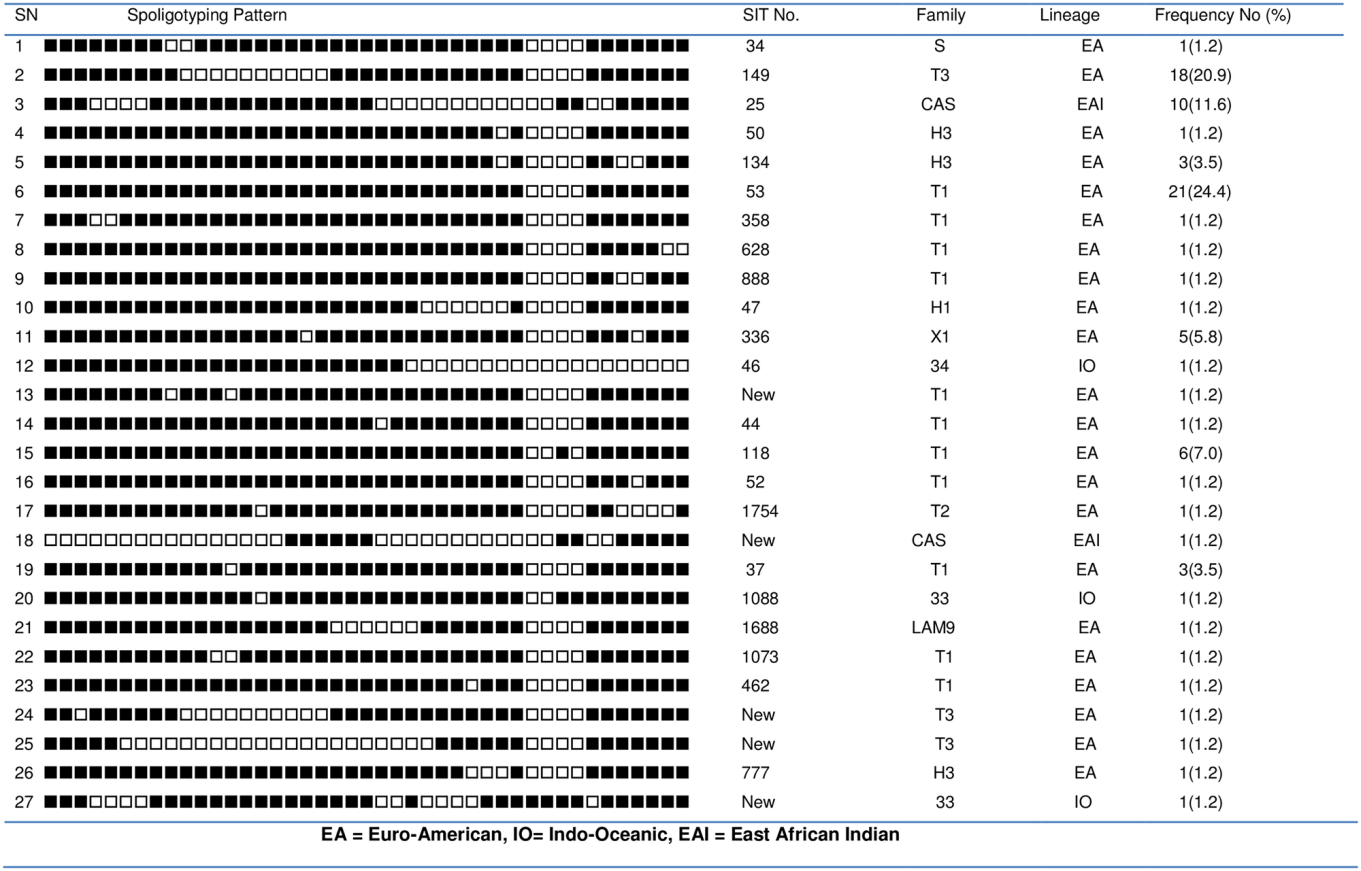
Spoligotype pattern of *M*. *tuberculosis* strains isolated from pulmonary tuberculosis patients in and around Ambo Town, Central Ethiopia, 2015. The black squares represent positive hybridization signals and white squares represent a lack of hybridization.

All the 86 isolates generated a PCR product and were identified as *M*. *tuberculosis* species by RD9 deletion typing. Spoligotyping led to the identification of 27 different patterns of which 22 had previously been reported. Five strains did not match with any known pattern in the international spoligotype data base. Most (76.7%) of the isolates were clustered into seven spoligotype patterns. The remaining strains belonged to single spoligotypes. The most predominant spoligotypes were SIT53, SIT149, and SIT25 consisting of 21, 18 and 10 isolates, respectively (Fig 1).

Assigning of the strains to the family using SPOTCLUST software revealed that 45.3%, 23.3%, 12.8% of the strains belonged to T1, T3, and CAS family, respectively. Furthermore, Haarlem 3and X1families each consisted of 4.7% of the isolates; while Family 33 consisted of 2.3%of the isolates.

The remaining isolates belonged to Haarlem 1, LAM9, T2, S, T4 and family 34 with each of these families consisting of 1.2% of the total isolates. Further assignment of the isolates into lineages using the SPOTCLUST software revealed that 83.7%of the isolates belonged to the Euro-American, 12.8% to East African Indian and 3.5% to Indo-Oceanic lineages.

### Resistance pattern to first line anti-TB drugs

Drug sensitivity test to the first line anti-TB drugs INH, RIF, ETB and to STM was carried out on a total of 86 *M*. *tuberculosis* isolates. Prevalence of any resistance to one of the drugs was 23.3%i.e. 76.7% the isolates were susceptible to all four anti-TB drugs. Any resistance to one drug was most frequently observed for INH (9.3%) of all isolates tested and ETB (7.0%) followed by STM (5.8%). Any resistance to rifampicin was found in only 1.2%. The highest proportion of mono resistance was observed against INH (4.7%) followed by ETB (2.3%) and STM (2.3%). There was no monoresistance to RIF (0%). Only a single isolate was found to be multi-drug resistant (MDR) (Table 2).

**Table 2:** Resistance pattern to first line anti-TB drugs among new smear positive pulmonary TB patients in and around Ambo Town, Central Ethiopia (n = 86), 2015.

Variables	Frequency, number (%)
**Any Resistance to one drug**	
Any INH	8(9.3)
Any RIF	1(1.2)
Any STM	5(5.8)
Any ETB	6(7.0)
**Resistant to only one drug**	
INH only	4(4.7)
RIF only	0(0)
STM only	2(2.3)
ETB only	2(2.3)
**Resistant to only two drugs**	
INH+ETB only	2(2.3)
INH+RIF only	0(0)
INH+STM only	1(1.2)
ETB+STM only	1(1.2)
**Resistant to four drugs**	
INH+RIF+STM+ETB	1(1.2)

Combined drug resistance among new TB patients was observed to INH + ETB only (2.3%), INH + STM only (1.2%) and ETB+STM only (1.2%). No drug resistance strain was identified for the combination of INH+RIF only. No triple resistance to first line anti-TB drugs was observed. One isolate (1.2%) was resistant to all four anti-TB drugs tested. Rifampicin resistance (RR) was observed in only one isolateamong86 isolates (1.2%) from newly diagnosed pulmonary TB patients (Table 2).

In this study, MDR-TB was detected in an HIV positive hospitalized study participant. It was not possible to investigate any association between HIV infection or hospitalization and the risk of MDR-TB because of the very low number of patients affected with the conditions.

## Discussion

In the present study, the strains of *M*. *tuberculosis* circulating in and around Ambo Town were identified and their drug sensitivity patterns described. The findings enrich the pool of available data on the epidemiology and drug sensitivity pattern of *M*. *tuberculosis* in the country. Identity and clustering patterns of *M*. *tuberculosis* strains were identified by RD9-PCR and spoligotyping. All of the isolates from sputum (86) were *M*. *tuberculosis* strains. The majority (66/86) of the isolates clustered into seven spoligotypes. Twenty spoligotypes were however unique consisting of only a single isolate each. According to the SITVIT database and spoligotyping International Typing (SIT) numbers the most prevalent shared types in the present study were SIT53 and SIT149. This is consistent with previous reports from Ethiopia. Belay *et al*.[15] reported SIT53 and SIT149 among the pastoral communities in the Afar Region of Ethiopia. Likewise, SIT 53 was the most frequent spoligotype in Dilla, southern Ethiopia [16]. Furthermore, SIT149 was reported to be the leading strain in Eastern Amhara [17]. The observation of a high degree of clustering and frequency of this *M*. *tuberculosis* spoligotype in the study area could suggest the epidemiological importance of these strains.

Further characterization of the strains to families using SPOTCLUST software revealed the predominance of three families, namely T1, T3 and CAS. The occurrence and distribution of these families varies from region to region in Ethiopia. Garedew *et al* [18] reported a high proportion of T1 and T3 family strains in Debre Berhan, central Ethiopia. Mihret *et al* [19] reported a high proportion of T and CAS family strains in Addis Ababa while, Derbew *et al* [20] reported a higher prevalence of T family strains in the southern region of Ethiopia. T1 and CAS families have similarly been reported to be frequent in other African countries such as Tanzania and Uganda [21, 22]. This indicates a possible broad distribution of these *M*. *tuberculosis* strains in East Africa.

Drug resistance among newly diagnosed pulmonary TB cases is a good epidemiological marker to trace the transmission of drug resistant strains in the community [23, 24]. In our study, the proportion of mono resistance to any one of the four drugs tested was 23.3%. This is relatively lower than in previous reports from different parts of the country [25]. For example, a relatively lower (18.4%) prevalence of mono resistance was reported by Abebe *et al* [26] from Jimma. Nevertheless, our finding is comparable with that of Seyoum *et al* [27] who reported 23%mono resistance from Eastern Ethiopia. The variation in overall prevalence of drug resistant TB among different study settings could be technical (such as due to variation in sample size, laboratory procedures, etc) and/or a true event (higher prevalence due to improper TB case management, delayed diagnosis, poor treatment follow up, poor treatment adherence and similar program failures).

In our study, the highest rate of monoresistance was observed against INH (4.7%). A relatively higher frequency of monoresistance to INH has been reported from Jimma (13.2%) and Eastern (9.5%) parts of the country [26, 27]. Lower rates (2.8%) of INH mono resistance among new pulmonary TB cases has been reported from northwest Ethiopia [28]. INH monoresistance recorded by the present study was comparable with those reported from other African countries such as Uganda (5.0%) [29] and Nigeria (3.8%) [30]. The relatively high proportion of INH resistance in several of the sites in Ethiopia could be due to the history of use of INH in the national TB control program over several decades. This condition may result in higher prevalence of MDR-TB if rifampicin resistance increases in frequency. We identified only one case of resistance to rifampicin and this was a strain resistant to all drugs tested. There was no monoresistance to the drug in our study. Although rifampicin resistance appears to be infrequent in the Ambo region, as it was in several places in Ethiopia in the past [31], the frequency of any resistance to RIF (RR) appears to be on the rise in Ethiopia. The prevalence of MDR TB among newly diagnosed pulmonary TB patients was only 1.6% until a few years back when the estimate was upgraded to 2.3% following the second national drug resistance prevalence survey. This was mainly because of the low frequency of resistance against rifampicin despite a higher frequency of INH resistance in the country. A 2012 study from northwest Ethiopia reported zero prevalence of RIF mono resistance but an MDR rate of 5%among 214 *M*. *tuberculosis* isolates from newly diagnosed pulmonary TB patients. Eight of the RR strains were also resistant to other anti-TB drugs (six to five, one to four and one to three) [28]. Relatively higher RIF monoresistance has been reported from southwestern and eastern parts of the country (0.7%, and 1.7% respectively) [26, 27]. The reason for the low RIF mono resistance could be to the relatively recent introduction of RIF into the anti-TB regimen and restricted access to the drug except for anti-TB treatment essentially in a combined fixed dose formulation.

In this study, the prevalence of MDR-TB among newly diagnosed pulmonary TB cases was found to be lower than the national prevalence rate of 2.3%. Previous reports of MDR-TB among newly diagnosed pulmonary TB cases rangedfrom0%to 3.7% in different study settings [26, 27,28, 32, 33]. The prevalence of MDR-TB varies over time and in different populations [29, 34]. This could be due to differences in sample size, methodology, or due to some specific characteristics of the studied population. The other possible reason could be due to differences in TB control program effectiveness. Implementation of active TB case finding and DST that leads to early detection and effective treatment of MDR-TB cases might limit the emergence and/or spread of MDR strains.

In this study association between HIV infection and risk of acquiring MDR-TB was not addressed. In different studies, discordant findings have been reported. Mesfin *et al* [35] conducted a systematic review and meta-analysis which concluded that a positive association was observed between HIV/AIDS and primary MDR-TB. In a similar review a positive association was observed between HIV infection and MDR-TB [36]. In general, an association of anti-TB drug resistance development and HIV infection could be explained by the fact that people living with HIV may also be more likely to be exposed to MDR-TB patients, due to increased hospitalization in settings with poor infection control. The other possible reason could be poor treatment adherence because HIV/AIDS patients may be subjected to a combination of various regimens including ART resulting in adverse events and unwillingness to continue regular medication. This situation may lead to accumulation of drug resistant strains.

## Conclusion and recommendation

This study provides the first evidence of genetic diversity of *M*. *tuberculosis* strains in the Ambo area of Central Ethiopia. The predominant strains circulating in the area seem to be similar to those dominant in different parts of the country (SIT53, SIT149, and SIT25; T1 family followed by T3 and CAS families). Although the prevalence of MDR-TB was low in this study, the high proportion of any resistance to first line drugs suggests that emergence of MDR-TB is probably delayed mainly because of the low resistance rate to rifampicin. This calls for close monitoring of drug resistance in health facilities with increased access to sensitivity testing.

## Supporting information

S1 File Raw data. (SAV)

## References

[1] World Health Organization. Global Tuberculosis Report 2016. www.who.int/tb/publications/global_report/en/. Accessed 1 May 2017.

[2] Minstry of Health, Federal Democratic Republic of Ethiopia. Guidelines on Programmatic Management of Drug Resistant Tuberculosis in Ethiopia. 2^nd^ Edition, 10 2014, Addis Ababa.

[3] Sougakoff W. Molecular epidemiology of multidrugresistant strains of Mycobacterium tuberculosis. Clinical Microbiology and Infection. 2011;17(6):800–5. doi: 10.1111/j.1469-0691.2011.03577.x 21682800 10.1111/j.1469-0691.2011.03577.x

[4] Magana-Arachchi DN. Epidemiology of Multidrug Resistant Tuberculosis (MDR-TB). 2013.

[5] Tessema B, Beer J, Merker M, Emmrich F, Sack U, Rodloff AC, et al Molecular epidemiology and transmission dynamics of Mycobacterium tuberculosis in Northwest Ethiopia: new phylogenetic lineages found in Northwest Ethiopia. BMC infectious diseases. 2013;13(1):1–11. 23496968 10.1186/1471-2334-13-131 PMC3605317

[6] Tessema B, Beer J, Emmrich F, Sack U, Rodloff AC. Analysis of gene mutations associated with isoniazid, rifampicin and ethambutol resistance among Mycobacterium tuberculosis isolates from Ethiopia. BMC infectious diseases. 2012;12(1):37. 22325147 10.1186/1471-2334-12-37 PMC3378438

[7] Abate D, Taye B, Abseno M, Biadgilign S. Epidemiology of anti-tuberculosis drug resistance patterns and trends in tuberculosis referral hospital in Addis Ababa, Ethiopia. BMC research notes. 2012;5(1):462. 22929063 10.1186/1756-0500-5-462 PMC3507648

[8] Hussein B, Debebe T, Wilder-Smith A, Ameni G. Drug susceptibility test on Mycobacterium tuberculosis isolated from pulmonary tuberculosis patients in three sites of Ethiopia. African Journal of Microbiology Research. 2013;7(9):791–6.

[9] World Health Organization. Global laboratory initiative advancing TB diagnosis. Mycobacteriology Laboratory Manual. Stop TB partnership; First Edition, April 2014.

[10] Parsons LM, Brosch R, Cole ST, et al Rapid and simple approach for identification of *Mycobacterium tuberculosis* complex isolates by PCR-based genomic deletion analysis. J ClinMicrobiol 2002; 40: 2339–2234. 10.1128/JCM.40.7.2339-2345.2002 PMC120548 12089245

[11] Kamerbeek J, Schouls L, Kolk A, Van Agterveld M, Van Soolingen D, Kuijper S, et al Simultaneous detection and strain differentiation of Mycobacterium tuberculosis for diagnosis and epidemiology. Journal of Clinical Microbiology. 1997;35(4):907–14. 9157152 10.1128/jcm.35.4.907-914.1997 PMC229700

[12] Shabbeer A, Cowan S L, Ozcaglar C, Rastogi N, vanDenberg SL, Yener B, Bennett KP. TB-Lineage. An online tool for classification and analysis of strains of Mycobacterium tuberculosis complex. Infect Genet Evol 2012; 12: 789–97. doi: 10.1016/j.meegid.2012.02.010 22406225 10.1016/j.meegid.2012.02.010

[13] World Health Organization Geneva (WHO). Guideline for surveillance of drug resistance in tuberculosis. 2003.

[14] Wedajo W, Schön T, Bedru A, Kiros T, Hailu E, Mebrahtu T, et al A 24-well plate assay for simultaneous testing of first and second line drugs against Mycobacterium tuberculosis in a high endemic setting. BMC research notes. 2014;7(1):512. 25108648 10.1186/1756-0500-7-512 PMC4267144

[15] Belay M, Ameni G, Bjune G, Couvin D, Rastogi N, Abebe F. Strain diversity of Mycobacterium tuberculosis isolates from pulmonary tuberculosis patients in afar pastoral region of Ethiopia. BioMed research international. 2014;2014. 10.1155/2014/238532 PMC3966356 24734230

[16] Gebrezgabiher G, Romha G, Ameni G. Spoligotyping of *M*. *tuberculosis* isolates from tuberculosis diagnosed patients at Dilla University Referral Hospital and other private clinics, Southern Ethiopia. Asian Pacific Journal of Tropical Disease. 2015;5(4):329–33.

[17] Esmael A, Wubie M, Desta K, Ali I, Endris M. Genotyping and Drug Resistance Patterns of M. tuberculosis in Eastern Amhara region, Ethiopia. J Clin Diagn Res. 2014;2:102.

[18] Garedew L, Mihret A, Mamo G, Abebe T, Firdessa R, Bekele Y, et al Strain diversity of mycobacteria isolated from pulmonary tuberculosis patients at Debre Birhan Hospital, Ethiopia. The International Journal of Tuberculosis and Lung Disease. 2013;17(8):1076–81. doi: 10.5588/ijtld.12.0854 23827032 10.5588/ijtld.12.0854

[19] Mihret A, Bekele Y, Aytenew M, Abebe M, Wassie L, Loxton G, et al Modern lineages of Mycobacterium tuberculosis in Addis Ababa, Ethiopia: implications for the tuberculosis control programe. African health sciences. 2013;12(3):339–44 10.4314/ahs.v12i3.15 PMC3557690 23382750

[20] Deribew A, Abebe G, Apers L, Abdissa A, Deribe F, Woldemichael K, et al Prevalence of pulmonary TB and spoligotype pattern of Mycobacterium tuberculosis among TB suspects in a rural community in Southwest Ethiopia. BMC Infectious Diseases. 2012;12(1):54. 22414165 10.1186/1471-2334-12-54 PMC3378444

[21] Mbugi EV, Katale BZ, Siame KK, Keyyu JD, Kendall SL, Dockrell HM, et al Genetic diversity of Mycobacterium tuberculosis isolated from tuberculosis patients in the Serengeti ecosystem in Tanzania. Tuberculosis. 2014. 10.1016/j.tube.2014.11.006 PMC4364622 25522841

[22] Lukoye D, Katabazi FA, Musisi K, Kateete DP, Asiimwe BB, Moses O, et al The T2 Mycobacterium tuberculosis Genotype, Predominant in Kampala-Uganda, Shows Negative Correlation with anti-Tuberculosis Drug Resistance. Antimicrobial agents and chemotherapy. 2014:AAC 02338–13. 10.1128/AAC.02338-13 PMC4068514 24777100

[23] Kamal M, Javaid A. Primary Drug Resistant Tuberculosis. Pakistan Journal of Chest Medicine. 2015;19(1).

[24] Villa-Rosas C, Laniado-Laborín R, Oceguera-Palao L. Primary drug resistance in a region with high burden of tuberculosis. A critical problem. Salud Pública de México. 2015;57(2). 10.21149/spm.v57i2.7414 26235779

[25] Getahun M, Ameni G, Kebede A, Yaregal Z, Hailu E, Medihn G, et al Molecular typing and drug sensitivity testing of Mycobacterium tuberculosis isolated by a community-based survey in Ethiopia. BMC public health. 2015;15(1):751. 26245282 10.1186/s12889-015-2105-7 PMC4527252

[26] Abebe G, Abdissa K, Abdissa A, Apers L, Agonafir M, de-Jong BC, et al Relatively low primary drug resistant tuberculosis in southwestern Ethiopia. BMC research notes. 2012;5(1):225. 22574696 10.1186/1756-0500-5-225 PMC3441821

[27] Seyoum B, Demissie M, Worku A, Bekele S, Aseffa A. Prevalence and Drug Resistance Patterns of Mycobacterium tuberculosis among New Smear Positive Pulmonary Tuberculosis Patients in Eastern Ethiopia. Tuberculosis research and treatment. 2014;2014. 10.1155/2014/753492 PMC4009208 24834351

[28] Tessema B, Beer J, Emmrich F, Sack U, Rodloff A. First-and second-line anti-tuberculosis drug resistance in Northwest Ethiopia. The International Journal of Tuberculosis and Lung Disease. 2012;16(6):805–11. doi: 10.5588/ijtld.11.0522 22390880 10.5588/ijtld.11.0522

[29] Lukoye D, Adatu F, Musisi K, Kasule GW, Were W, Odeke R, et al Anti-Tuberculosis Drug Resistance among New and Previously Treated Sputum Smear-Positive Tuberculosis Patients in Uganda: Results of the First National Survey. PloS one. 2013;8(8):e70763 doi: 10.1371/journal.pone.0070763 23936467 10.1371/journal.pone.0070763 PMC3731251

[30] Uzoewulu N, Ibeh I, Lawson L, Goyal M, Umenyonu N. Drug Resistant Mycobacterium tuberculosis in Tertiary Hospital South East, Nigeria. J Med Microb Diagn. 2014;3(141):2161–0703.1000141.

[31] Weldegebreal S. and Mebrahtu T. (2017). "Anti-tuberculosis drug resistance in Ethiopia: systematic review." The International Journal of Tuberculosis and Lung Disease 21(1): 18–22. doi: 10.5588/ijtld.16.0286 28157460 10.5588/ijtld.16.0286

[32] Adane K, Ameni G, Bekele S, Abebe M, Aseffa A. Prevalence and drug resistance profile of Mycobacterium tuberculosis isolated from pulmonary tuberculosis patients attending two public hospitals in East Gojjam zone, northwest Ethiopia. BMC public health. 2015;15(1):572. 26092570 10.1186/s12889-015-1933-9 PMC4473837

[33] Maru M, Mariam SH, Airgecho T, Gadissa E, Aseffa A. Prevalence of Tuberculosis, Drug Susceptibility Testing, and Genotyping of Mycobacterial Isolates from Pulmonary Tuberculosis Patients in Dessie, Ethiopia. Tuberculosis Research and Treatment. 2015;2015:1–10. 10.1155/2015/215015 PMC4477223 26180642

[34] Massi M, Wahyuni S, Halik H, Yusuf I, Leong F, Dick T, et al Drug resistance among tuberculosis patients attending diagnostic and treatment centres in Makassar, Indonesia. The International Journal of Tuberculosis and Lung Disease. 2011;15(4):489–95. doi: 10.5588/ijtld.09.0730 21396208 10.5588/ijtld.09.0730

[35] Mesfin YM, Hailemariam D, Biadglign S, Kibret KT. Association between HIV/AIDS and multi-drug resistance tuberculosis: a systematic review and meta-analysis. PloS one. 2014;9(1):e82235 doi: 10.1371/journal.pone.0082235 24416139 10.1371/journal.pone.0082235 PMC3885391

[36] Suchindran S, Brouwer ES, Van Rie A. Is HIV infection a risk factor for multi-drug resistant tuberculosis? A systematic review. PLoS One. 2009;4(5):e5561 doi: 10.1371/journal.pone.0005561 19440304 10.1371/journal.pone.0005561 PMC2680616

